# Myelinosome Organelles in the Retina of R6/1 Huntington Disease (HD) Mice: Ubiquitous Distribution and Possible Role in Disease Spreading

**DOI:** 10.3390/ijms222312771

**Published:** 2021-11-25

**Authors:** Marina G. Yefimova, Emile Béré, Anne Cantereau-Becq, Annie-Claire Meunier-Balandre, Bruno Merceron, Agnès Burel, Karine Merienne, Célia Ravel, Frédéric Becq, Nicolas Bourmeyster

**Affiliations:** 1 Laboratoire Signalisation et Transports Ioniques Membranaires, Université de Poitiers/CNRS, 1 Rue Georges Bonnet, 86022 Poitiers, France; anne.becq@univ-poitiers.fr (A.C.-B.); annie-claire.balandre@univ-poitiers.fr (A.-C.M.-B.); frederic.becq@univ-poitiers.fr (F.B.); nicolas.bourmeyster@univ-poitiers.fr (N.B.); 2 Sechenov Institute of Evolutionary Physiology and Biochemistry, Russian Academy of Sciences, 44 Pr. Thorez, 194233 St. Petersburg, Russia; 3 Laboratoire de Biologie de la Reproduction-CECOS, Hopital SUD, 16 Bd de Bulgarie, CEDEX, 35000 Rennes, France; Celia.RAVEL@chu-rennes.fr; 4 Plateforme IMAGE-UP, 1 Rue Georges Bonnet, 86022 Poitiers, France; emile.bere@univ-poitiers.fr (E.B.); bruno.merceron@univ-poitiers.fr (B.M.); 5 Plateforme Mric TEM, BIOSIT UMS 34 80, Université de Rennes 1, 2 Av. Pr. Léon Bernard, CEDEX, 35043 Rennes, France; agnes.burel@univ-rennes1.fr; 6 Laboratory of Cognitive and Adaptive Neurosciences (LNCA), University of Strasbourg, 67000 Strasbourg, France; karine.merienne@unistra.fr; 7 CNRS UMR 7364, 67000 Strasbourg, France; 8 Service de Biochimie, CHU de Poitiers, 1, Rue de la Milétrie, 86021 Poitiers, France

**Keywords:** myelinosomes, retina, glial Müller cells, mutant Huntingtin, Huntington disease, membrane fusion, CAG expansion, neurodegeneration, Huntington disease biomarker, Huntington disease spreading

## Abstract

Visual deficit is one of the complications of Huntington disease (HD), a fatal neurological disorder caused by CAG trinucleotide expansions in the *Huntingtin* gene, leading to the production of mutant Huntingtin (mHTT) protein. Transgenic HD R6/1 mice expressing human HTT exon1 with 115 CAG repeats recapitulate major features of the human pathology and exhibit a degeneration of the retina. Our aim was to gain insight into the ultrastructure of the pathological HD R6/1 retina by electron microscopy (EM). We show that the HD R6/1 retina is enriched with unusual organelles myelinosomes, produced by retinal neurons and glia. Myelinosomes are present in all nuclear and plexiform layers, in the synaptic terminals of photoreceptors, in the processes of retinal neurons and glial cells, and in the subretinal space. In vitro study shows that myelinosomes secreted by human retinal glial Müller MIO-M1 cells transfected with *EGFP-mHTT-exon1* carry EGFP-mHTT-exon1 protein, as revealed by immuno-EM and Western-blotting. Myelinosomes loaded with mHTT-exon1 are incorporated by naive neuronal/neuroblastoma SH-SY5Y cells. This results in the emergence of mHTT-exon1 in recipient cells. This process is blocked by membrane fusion inhibitor MDL 28170. Conclusion: Incorporation of myelinosomes carrying mHTT-exon1 in recipient cells may contribute to HD spreading in the retina. Exploring ocular fluids for myelinosome presence could bring an additional biomarker for HD diagnostics.

## 1. Introduction

The retina is a part of the CNS, exhibiting similar principles of cellular organization and metabolism [1]. Several major neurodegenerative disorders such as Parkinson’s and Alzheimer’s disease or multiple sclerosis have manifestations in the eye. Various eye-specific pathologies share characteristics of other CNS pathologies. Furthermore, ocular manifestations precede the symptoms in the brain, offering the opportunity of early diagnosis of neurodegenerative diseases in human and animal models [1].

Visual deficit is one of the complications of Huntington disease (HD)—a fatal autosomal-dominant late-onset neurological disorder that causes progressive and irreversible motor dysfunctions, resulting in coordination and gait difficulties, as well as cognitive and behavioral changes [2]. Orthostatic hypotension, excessive perspiration, tachycardia, and vegetative symptoms are prominent in HD (cited from [2]). Degeneration and neural loss of the striatum, particularly the caudate nuclei, targeting the cerebral cortex, pallidum, thalamus, brainstem, and cerebellum, are specific neuropathological findings in HD (cited from [2]) The degree of the pathological changes correlates with that of disability. HD is characterized by neuroinflammation caused by microglial activation, so that the corresponding inflammatory markers are significantly increased in the plasma of HD individuals [3]. A post-mortem study reveals increased expression of inflammatory mediators in the cortex and the cerebellum [4]. HD also manifests multiple abnormalities in peripheral organs such as the heart, skeletal muscle, thyroid, and the digestive and reproductive tracts [5,6]. Effective treatments to halt disease before the onset of disabling symptoms are still unavailable [7], but education and symptomatic therapies are the current tools for clinicians to use with patients and families affected by HD [8,9]. The epidemiology of HD reveals gross differences in the prevalence of HD by ancestry, with a much higher rate of the disease in populations of European descent, which varies between 1.6 and 12.3 cases per 100,000 [10]. In this context, there is an unmet need to identify preclinical biomarkers to select the high-risk population and predict the disease and its progression [11].

HD belongs to the class of repeat expansion diseases [12]. It is caused by the expansion of CAG triplets in the gene coding for ubiquitously expressed protein Huntingtin (*HTT*), a large monomer of 350 kDa [13]. CAG expansion (above 37–40 repeats) in the exon1 of *HTT* results in the generation of an abnormally long polyglutamine track (polyQ) in the N-terminus of the protein [14] that perturbs protein properties and makes it prone to aggregation. The severity of HD phenotype correlates with the length of CAG blocks [12]. Mutant HTT (mHTT) is associated with ballooning cell death (BCD) in the CNS. BCD is triggered in a mechanism of transcriptional repression-induced atypical cell death of neuron (TRIAD) with reduced levels of a transcriptional co-activator yes-associated protein (YAP) and transcriptional enhancer factor (TEF) [15]. Evidence has accumulated that mHTT can spread throughout the CNS in a prion-like fashion, as occurs in Parkinson’s and Alzheimer’s disease and amyotrophic lateral sclerosis [16]. Transneuronal spreading of mHTT is considered an important contributor to non-cell autonomous damage of brain networks in HD [16].

Key changes to the visual system, including retinal thinning, temporal retinal nerve fiber layer thinning, loss of retinal ganglion cells, reduced visual evoked potentials, impaired color vision, and poor motion perception, have been evidenced in patients suffering from HD [17,18,19,20]. Retinal dysfunction and degeneration were evidenced in rodent and *Drosophila* models of HD [21,22,23,24,25,26]. In both fly and rodents, retinal degeneration was progressive and dependent on CAG length [19,21]. Transgenic R6/1 and R6/2 mouse lines expressing human polyQ-expanded Htt exon 1 (115 and 150 CAG repeats, respectively) under human HTT promoter have proved to be the most popular models to study a mild late-onset or severe juvenile forms of HD, respectively. Compared to R6/1 mice, R6/2 mice manifest the accelerated form of the disease and a more severe phenotype [22,23]. Electron microscopy (EM) examination of R6/2 retina revealed a strong degeneration of the outer retina, while the inner retina was rather preserved.

Transgenic R6/1 mice recapitulate well enough a late-onset human pathology and exhibit prolonged longevity (>1 year) compared to R6/2 and other HD model mice [27,28,29]. The resulting level of transgene expression in R6/1 mice is 31% of the endogenous HTT [27,28]. The retinal phenotype was observed to occur at the same time as other neurological deficits, such as motor dysfunction (by 13 weeks of age) in the disease process. A specific functional deficit in cone response to the electroretinogram is thought to be due to a progressive and complete loss of cone opsin and transducin protein expression by 20 weeks of age [25]. Exploring histological sections showed the ‘wavy’ aspect of the degenerative R6/1 retina without the extensive cell loss [22,25]. Immunohistochemical study revealed the stress of retinal glia, estimated using anti-glial fibrillary acid protein (GFAP) labeling of glial Müller cells [25]. Nevertheless, information regarding the ultrastructure of the HD R6/1 retina was still missing.

In many aspects of tissue homeostasis maintenance, the retina shares striking similarities with the testis [30,31,32]. This concerns the isolation of both tissues from the bloodstream [33,34], the immune privilege [35,36], the cyclic character of main physiological processes (circadian rhythm in the retina) [37] and seminiferous epithelium cycle in the testis [38], a similar mode of apoptotic substrate cleaning [32,39,40], and a similar fatty acid composition of cell membranes [41,42].

We recently demonstrated that HD R6/1 testis produced rare organelles myelinosomes loaded with mHTT-exon1 [43,44]. Being secreted by testis somatic Sertoli cells, myelinosomes protected them from the accumulation of the toxic mHTT-exon1 protein in their cytoplasm [43,44]. Myelinosomes were described years ago as rare organelles, observed in a variety of cells under pathological situations caused by genetic or environmental factors [45]. The term “myelinosomes” was assigned to these organelles by electron microscopy investigators. Invisible by light microscopy, myelinosomes have, in EM micrographs, a myelin sheath structure consisting of stacked electron-dense osmiophile membranes, enwrapping the cavity filled with an electron lucid matrix [43]. Nevertheless, myelinosome vesicles were found in various extra-CNS tissues devoid of myelin, including testis seminiferous tubules, and others [43,45] (myelinosome vesicles are unusual organelles, but they are not the result of oligodendrocyte damage causing the formation of local myelin out-foldings, also termed “myelinosomes” [46]).

We aimed this study at characterizing the ultrastructure of HD R6/1 mice retina and to seek out the presence of myelinosomes in this tissue. Here we demonstrate, for the first time, that HD R6/1 retina is enriched with myelinosome organelles. In pathological R6/1 retina, both neuronal and glial cells contribute to the retinal pool of myelinosomes, which are detected in all nuclear and plexiform layers, in the synaptic terminals of photoreceptors, and in the processes of retinal neurons and glial cells, as well as in the subretinal space. Myelinosomes released by retinal glial Müller cells transfected with *EGFP-mHTT-exon1* are loaded with EGFP-mHTT-exon1 protein. Therefore, we also aimed to assess a plausible impact of myelinosomes carrying mutant protein on the neighboring cells. We demonstrate in vitro that myelinosomes carrying mHTT-exon1 are incorporated by human neuronal/neuroblastoma SH-SY5Y cells through membrane fusion mechanism. Thus, providing the transfer of mHTT–exon1 from glial Müller cells through interaction with neurons, myelinosomes may contribute to the spreading of the mHTT-exon1 protein in the retina.

## 2. Results

### 2.1. Transgenic R6/1 Mice Retina Undergoes Remodeling without Cell Loss

In our study, we used 23 postnatal (PN) week-old animals. This age corresponds to the middle symptomatic stage of the pathology in both the brain and the retina [25,28,29].

**Figure 1:**
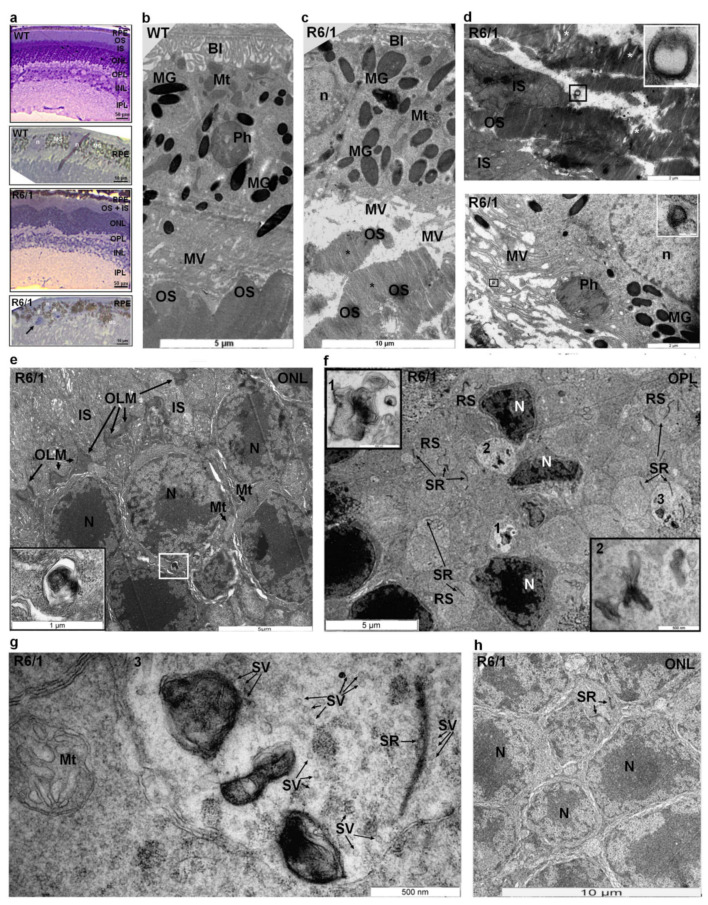
EM analysis of the outer retina of HD R6/1 mice. (**a**) Histological sections of eyes from 23-week-old normal and R6/1 mice. The two top panels are the sections of retina and RPE from normal mice, the two bottom panels are the sections of retina and RPE from R6/1 mice. Note the wavy aspect of the outer nuclear layer (ONL), disorganization of photoreceptor segment layers ((OS) + (IS)), misplacement of melanin granules (MG) in the RPE layer, and the presence of ectopic cells in the subretinal space (arrow, bottom panel) in R6/1 mice. (**b**,**c**) Electron micrograph of the fragment of outer retina of control (**b**) and R6/1 (**c**) mice. Note the loss of adjacency of OS tips to RPE cells, which display short, disorganized microvilli (MV). The density of melanin granules (MG) seems more pronounced in R6/1 retinas. (**d**) The subretinal space of the R6/1 retina contains fragments of swollen OS (top panel, white asterisks) and myelinosome vesicle (square selection, magnification is in the top right corner) between the fragments of photoreceptor inner (IS) and outer (OS) segments. In the bottom panel, long microvilli of RPE cells engulf a fragment of OS, forming a primary phagosome (Ph). Myelinosome (square selection, magnification is in the top right corner) localizes between the microvilli of RPE cells. (**e**) Electron micrograph of the R6/1 ONL. Note the fragments of IS retracted into the ONL, located below the outer limiting membrane (OLM). Some photoreceptor nuclei are surrounded with large cytoplasmic compartments, containing the mitochondria (Mt) and myelinosome organelle (selection, left bottom corner). (**f**) The OPL of R6/1 mice retina contains the rod spherules (RS), which harbors synaptic ribbons (SR). Note the presence of electron-dense myelinosome membranes (1, 2, 3) next to displaced photoreceptor nuclei (N); (**g**) is the selection 3 from (**f**). Rod spherule containing synaptic ribbon, which holds the synaptic vesicles. Note that some synaptic vesicles are adjacent to electron-dense membranes; (**h**) shows a displaced synaptic ribbon in the R6/1 ONL. Abbreviations: BI—basal infoldings; IS—photoreceptor inner segment; MG—melanin granules; Mt—mitochondrion; MV—microvilli; n, N—nucleus; OLM—outer limiting membrane; ONL—outer nuclear layer; OPL—outer plexiform layer; OS—photoreceptor outer segment; Ph—phagosome; RPE—retinal pigmented epithelium; RS—ribbon synapses, SV—synaptic vesicles.

In agreement with literature data [22,25], we also detected various histological abnormalities in R6/1 retinal sections compared to the control (Figure 1a). The wavy aspect of the outer nuclear layer (ONL), disorganization of the photoreceptor outer segment (OS) layer (Figure 1a, bottom panels), and the presence of displaced cells in the subretinal space (Supplementary Materials Figure S1a,b) were the hallmarks of 23 PN week-old R6/1 retinas. Furthermore, the retinal pigmented epithelium (RPE) layer in R6/1 retinal sections also looked disorganized compared to the control (Figure 1a, bottom panel; Supplementary Materials Figure S1a). No extensive cell loss was observed in the retinal sections from 23 PN week-old HD mice, but only rare apoptotic nuclei we detected in the ONL (Supplementary Materials Figure S1c). This is in agreement with literature data [22,25].

EM analysis of 23 PN week-old outer retina of HD R6/1 mice corroborated histological observations (Figure 1a,c–h). By this age, the degeneration of the photoreceptor OS layer was prominent. In contrast to control retina (Figure 1b), a tight structural adjacency of OS tips to the apical side of RPE cells was lost in R6/1 mice (Figure 1c,d, Supplementary Materials Figure S1a,b). Nevertheless, some photoreceptor OS looked rather preserved, consisting of tightly packed photoreceptor membranes (discs) (Figure 1c, black asterisks), while others were of irregular shape, were swollen, and presented less densely packed discs (Figure 1d, top panel, white asterisks; Supplementary Materials Figure S1b).

The cells from the RPE layer of R6/1 mice retina also displayed heterogeneity compared to control retinas (Figure 1a, bottom panel; Supplementary Materials Figure S1a,b). In R6/1 retinal sections, we detected the RPE cells possessing long microvilli that engulfed the fragments of photoreceptor discs (Figure 1d, bottom panel), suggesting the ability of cells to phagocyte the tips of OS, but not the ectopic cells we noticed in the subretinal space (Supplementary Materials Figure S1b). We also observed the cells exhibiting only short irregular microvilli (Figure 1c, Supplementary Materials Figure S1b). On the contrary, the basal infoldings of R6/1 RPE cells looked intact compared to the control (Figure 1b,c). The distribution of melanin granules in R6/1 RPE was less homogenous (Figure 1a, bottom panel, Supplementary Materials Figure S1a) than in control RPE (Figure 1a, top panels). Indeed, we often detected the RPE cells enriched with melanin granules, which burned into the subretinal space of R6/1 retinas (Figure 1a, bottom panel, Supplementary Materials Figure S1a).

The integrity of the photoreceptor IS layer was also perturbed in 23 PN week-old R6/1 retinas. Thus, occasionally, the IS located above the outer limiting membrane (OLM) were not tightly adjacent (Supplementary Materials Figure S1c). In some cases, the cytoplasm of IS was abnormally retracted in the outer nuclear layer (ONL), containing the nuclei of photoreceptors (Figure 1e). In these cells, the large mitochondria were discerned in close proximity to photoreceptor nuclei (Figure 1e), located below the OLM, which appeared intact (Figure 1e, Supplementary Materials Figure S1c). On very rare occasions, the apoptotic nuclei were found in the R6/1 ONL but not in the control retinas (Supplementary Materials Figure S1c,d).

We also often detected the displaced photoreceptor nuclei in the outer plexiform layer (OPL) of the degenerative HD retina (Figure 1f, Supplementary Materials Figure S1e). In non-pathologic retina, the photoreceptor nuclei are absent from the OPL, which harbors the synaptic contacts of photoreceptor cells with retinal interneurons. The later contains the ribbon synapses characterized by the presence of an electron-dense synaptic ribbon that holds synaptic vesicles [47] (Supplementary Materials Figure S2a). Photoreceptor ribbon synapses with the tips of horizontal cell axons and the dendrite tips of rod bipolar cells invaginate into the rod spherule (RS). In 23 PN week-old HD R6/1 retinas, the structurally normal electron-dense ribbon synapses, holding the synaptic vesicles in RS cytoplasm, were detected throughout the OPL (Figure 1f,g) as well as the ribbon synapses from cone photoreceptors, invaginating into the cone pedicles (CP) (Supplementary Materials Figure S1e). Nevertheless, the displaced ribbon synapses were detected in the depths of the R6/1 ONL (Figure 1h), suggesting the extension of neurites from retinal second-order neurons into the layer of photoreceptor nuclei. This corroborated previous immuno-histochemical study [25].

**Figure 2:**
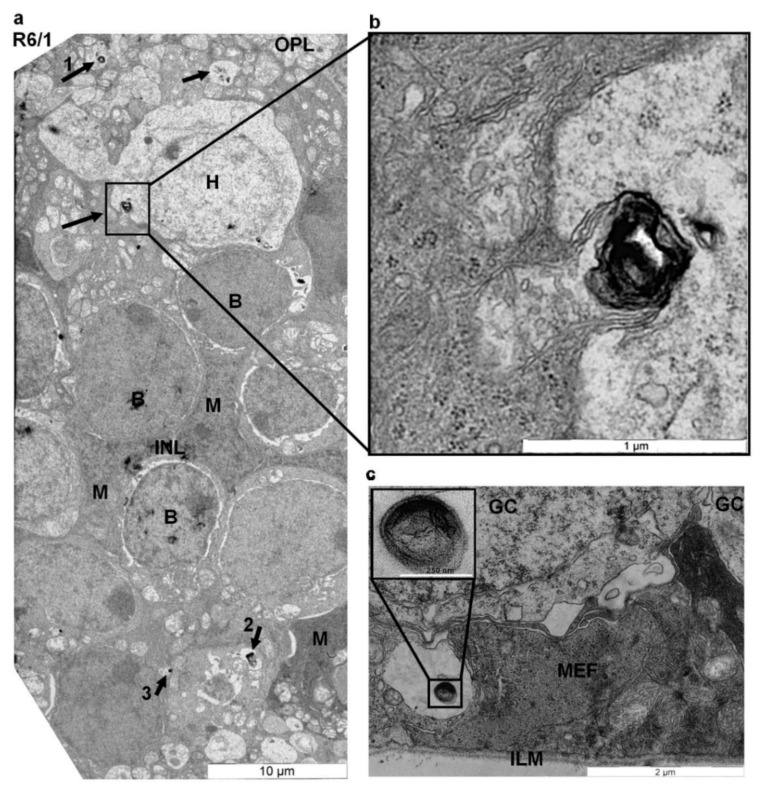
EM analysis of the inner retina of HD R6/1 mice. (**a**,**b**) Electron micrograph of the INL from 23-week-old R6/1 mice. (**b**) is a selection from (**a**). Cell nuclei in the INL are identified according to [50]. Myelinosomes (black arrows) are enclosed in vacuoles or discharged from cells displaying an osmiophilic cytoplasm and dark elongated nuclei (**a**,**b**), which is a characteristic of glial Müller cells (M). Magnifications of myelinosomes 1, 2, and 3 are in Supplementary Materials Figure S1f; (**c**) is the innermost part of the retina, showing two ganglion cell nuclei separated by the processes of Müller cells. Müller cell end-feet (MEF) contribute to the formation of the inner limiting membrane (ILM). Selection in (**c**) is myelinosome organelle located in vacuole next to the ILM. Abbreviations: A—amacrine cell; B—bipolar cell; BV—blood vessel; H—horizontal cells; GC—ganglion cell; ILM—inner limiting membrane; INL—inner nuclear layer; M—Müller cell; MEF—Müller cell end-feet.

Compared to the outer retina, the inner retina of 23 PN week-old HD R6/1 mice presented no apparent morphological abnormalities. Thus, the nuclei of the interneurons forming the inner nuclear layer (INL) as well as the nuclei of glial Müller cells were clearly detected by EM in both control and HD retinas (Figure 2a, Supplementary Materials Figure S2b–e). No morphological signs of apoptosis [48] were detected in the INL of R6/1 retina (Figure 2a; Supplementary Materials Figure S2b–e). The inner plexiform layer (IPL) of 23 PN week-old HD R6/1 also looked preserved and contained the ribbon synapses (Supplementary Materials Figure S2g) among a bipolar axon and the postsynaptic processes from amacrine or ganglion cells, located in the ganglion cell layer (GCL) [49]. Likewise, as in control retinas (Supplementary Materials Figure S2h), the ganglion cells from 23 PN week-old HD R6/1 retinas were separated from each other by glial processes of Müller cells (Figure 2c). The inner limiting membrane (ILM), formed by the astrocytes and the end-feet of Müller cells, also looked well preserved in HD R6/1 retina (Figure 2c).

### 2.2. Myelinosome Organelles Are Present in All Layers of R6/1 Mice Retina

Throughout all retinal thicknesses of the R6/1 mice, we encountered unusual electron-dense multi-stacked membrane structures, whose size varied from 120 to 700 nm, so that the average size was 470.0 ± 18.5 nm. By morphological criteria, these structures corresponded to myelinosomes, which are rare organelles of unknown origin [45]. In R6/1 retinal sections, myelinosomes were recognized as electron dense osmiophilic vesicles, consisting of multiples membrane stacks enwrapping an electron-lucid matrix. Basically, myelinosomes from R6/1 retinas strongly looked like those we previously described in R6/1 testis [43].

In the retina of R6/1 mice, myelinosome organelles were present in all nuclear and all plexiform layers, and in the subretinal space (Figure 1d–g, Figure 2, Supplementary Materials Figure S1e,f). In the outer retina of R6/1 mice, myelinosome organelles were encountered in perinuclear cytoplasm (Figure 1e) or in the synaptic terminals of rod and cone photoreceptors (Figure 1f,g; Supplementary Materials Figure S1e). The vacuoles harboring myelinosomes were detected throughout the OPL, wherein Müller cell processes envelop groups of neural processes (Figure 2a; Supplementary Materials Figure S1e,f). Myelinosomes were also distinguished in the subretinal space, suggesting the liberation of these organelles from retinal cells (Figure 1d). Of note is that no myelinosomes were found in the proximity of the sporadic apoptotic nuclei we detected in the R6/1 ONL (Supplementary Materials Figure S1c).

In the inner retina, myelinosomes were present in perinuclear cytoplasm of retinal interneurons, in extracellular spaces inside the INL and in glial Müller cells, whose nuclei are also located in the INL (Figure 2a; Supplementary Materials Figures S1f and S2b–f). By EM analysis, we were able to detect myelinosome vesicles leaving Müller cell cytoplasm. As shows Figure 2a,b, this occurs after fusion of myelinosome-bearing vacuole with the plasma membrane of the Müller cell. Electron-dense myelinosomes were also found inside the IPL in neuronal processes formed by the dendrites of ganglion cells and by the axons of retinal interneurons (Supplementary Materials Figure S2g), as well as in the GCL in close proximity to the ILM, separating the retina from the vitreal space (Figure 2c). No myelinosomes were detected in all retinal layers from the control retinas (Figure 1b; Supplementary Materials Figures S1d and S2a,h).

Thus, EM data indicate that myelinosome organelles are present in both neuronal and glial cell populations of HD retina. It was then of interest to gain insight into the putative role of these organelles in the pathological R6/1 retina.

### 2.3. Myelinosomes Are Released from MIO-M1 Müller Cells Transfected with EGFP-mHTT-exon1

**Figure 3:**
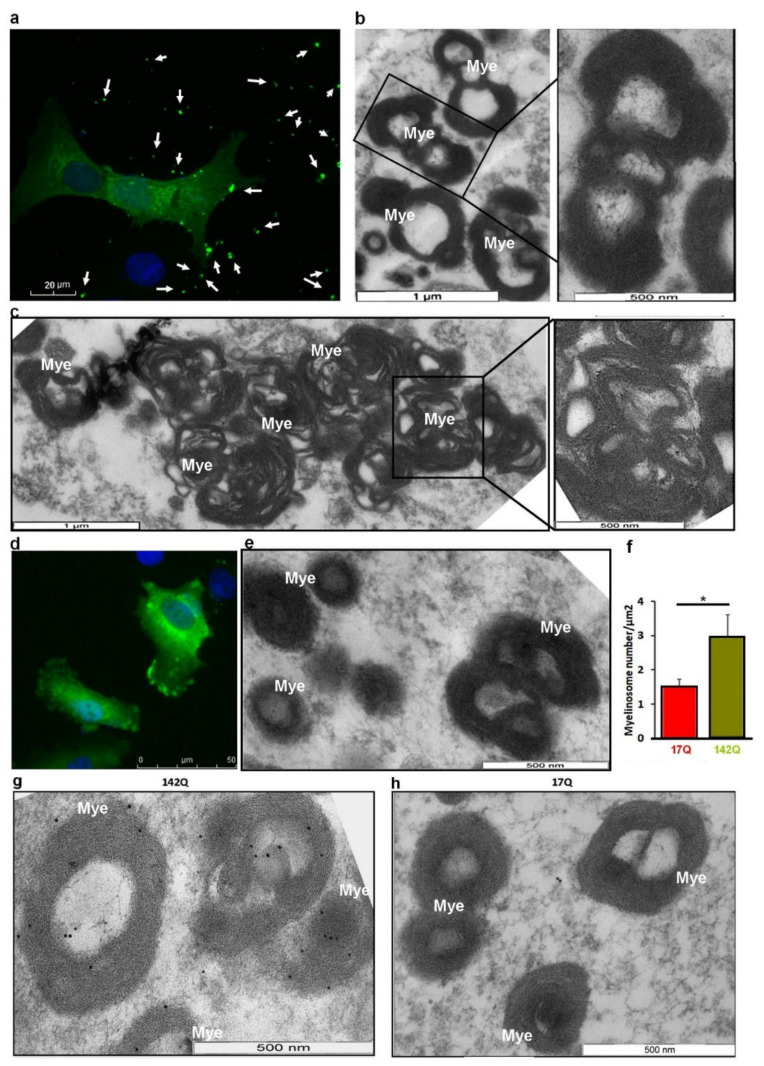
MIO-M1 Müller cells produce myelinosomes. (**a**) IF pictures of MIO-MI cells expressing *EGFP-mHTT-exon1* 48 h post-transfection. Arrows denote green-colored extracellular spots in the vicinity of cells. (**b**) Myelinosome vesicles isolated from 20,000 g culture media pellets of MIO-M1 Müller cells expressing *EGFP-mHTT-exon1.* Right panel is a magnification of a selection from the left panel. (**c**) Agglomerated myelinosomes from MIO-M1 Müller cells expressing *EGFP-mHTT-exon1.* Note the “loose” aspect of myelinosomes, probably due to their immature state. Right panel is a magnification of a selection from the left panel, showing the decreased density of osmiophile membrane packaging. (**d**) IF pictures of MIO-MI cells expressing *EGFP-HTT-exon1* 48 h post-transfection. Note the absence of extracellular green staining. (**e**) Myelinosomes isolated from 20,000 g culture media pellets of MIO-M1 Müller cells expressing *EGFP-HTT-exon1.* Right panel is a magnification of a selection from the left panel. (**f**) Histogram representing quantification of myelinosomes isolated from the preparations of 20,000 g culture media pellet of MIO-M1 Müller cells expressing EGFP-HTT-exon1 (17Q) and EGFP-mHTT-exon1 (142Q) protein.* *p* < 0.05 by two-tailed Student’s *t* test. (**g**,**h**) Immunogold labeling of 20,000 g culture media pellet of MIO-M1 Müller cells transfected with *EGFP-mHTT-exon1* (**g**) and *EGFP-HTT-exon1* (**h**) with anti-EGFP antibody. Note that gold particles are only associated with myelinosomes from MIO-M1 Müller cells transfected with *EGFP-mHTT-exon1* (**g**). Abbreviations: Mye—myelinosomes.

EM examination of retinal sections showed that, in situ, the retinal glial Müller cells produced and secreted the myelinosomes (Figure 2a,b).Therefore, to ascertain a plausible role of myelinosomes in pathological HD R6/1 retinas, in our subsequent in vitro experiments we used the MIO-M1 human retinal glial Müller cell line [51]. We transiently transfected MIO-M1 cells with a plasmid vector carrying *EGFP-mHTT-exon1.* Figure 3a shows that EGFP-mHTT-exon1 staining was present in cell cytoplasm and in small vesicles of transfected MIO-M1 cells. Furthermore, we observed numerous green-colored spots in the extracellular spaces of MIO-M1 cells expressing EGFP-mHTT-exon1 (Figure 3a). We then aimed to examine the morphology of the released particles.

Forty-eight hours post-transfection, we collected the culture media of MIO-M1 cells expressing EGFP-mHTT-exon1, processed it through differential centrifugation technique we had previously developed for myelinosome isolation (see Math & Meth [43]), and then analyzed the ultrastructure of the pellets. EM analysis revealed a large set of multi-stacked electron-dense osmiophile vesicles released from MIO-M1 cells, expressing mutant EGFP-mHTT-exon1 protein (Figure 3b,c). As in myelinosomes from R6/1 retinas (Figure 1d–g and Figure 2a–c; Supplementary Materials Figures S1e,f and S2b–g), MIO-M1-derived myelinosomes showed variability with respect to their size (from 100 to 800 nm), shape (round-shaped, elliptic, multiconcentric), discernibility of electron-lucid matrix, and density of membrane packaging (Figure 3b,c). In some cases, myelinosomes vesicles were agglomerated in large packs, wherein the majority of myelinosomes looked “loose” owing to the decreased density of osmiophile membrane packaging (Figure 3c), probably because of their immature state. EM analysis also revealed intracellular myelinosomes in the cytoplasm of MIO-M1 Müller cells, transfected with *EGFP-mHTT-exon1*, but not in untransfected ones (Supplementary Materials Figure S3a,b).

In control experiments, we transfected the glial MIO-M1 cells with plasmid vector carrying normal *EGFP-HTT-exon1*. In agreement with literature data [52], EGFP-HTT-exon1 protein was present in cell cytoplasm and in small vesicles. No extracellular green spots were detected in this case (Figure 3d). Nevertheless, after differential centrifugation of the culture media, EM analysis revealed myelinosome organelles in culture media pellet from MIO-M1 cells, expressing EGFP-HTT-exon1 (Figure 3e). A quantification study showed that myelinosomes emitted by control cells were less numerous compared to myelinosomes released by MIO-M1 cells expressing EGFP-mHTT-exon1 (1.48 ± 0.22 vesicle/μm^2^ (*n* = 36) vs. 2.89 ± 0.68 vesicle/μm^2^ (*n* = 19), respectively) (Figure 3f). Of note is that myelinosome-containing fractions from retinal glial MIO-M1 Müller cells expressing either *EGFP-mHTT-exon1* or *EGFP-HTT-exon1* were free of any other components (organelles or vesicles) (Figure 3b,c,f,e).

We previously demonstrated in situ and in vitro that myelinosomes produced by HD R6/1 testis or by Sertoli cells transfected with *EGFP-mHTT-exon1*, were loaded with mHTT-exon1 protein [43]. Therefore, by immuno-EM technique, we checked for the presence of the EGFP-mHTT-exon1 protein in myelinosome preparations using anti-EGFP antibodies [53]. Figure 3g shows that myelinosomes released from MIO-M1 cells expressing EGFP-mHTT-exon1 contained gold particles on their membranes, attesting to the presence of the EGFP-mHTT-exon1 protein. On the contrary, myelinosomes emitted from MIO-M1 cells expressing EGFP-HTT-exon1 did not contain gold granules on their surface (Figure 3h). Some rare gold particles detected in the preparation did not exhibit any precise localization, showing the slight background noise of the technique (Figure 3h). This corroborated our previous data showing no association of normal EGFP-HTT-exon1 protein with myelinosomes [43]. Thus, immuno-EM analysis demonstrated that myelinosome vesicles released from human retinal glial MIO-M1 Müller cells transfected with *EGFP-mHTT-exon1* were loaded with the mutant EGFP-mHTT-exon1 protein. It was then of interest to assess whether these myelinosomes could interact with neighboring cells.

### 2.4. Myelinosomes Are Incorporated into Human Neuroblastoma SH-SY5Y Cells

**Figure 4:**
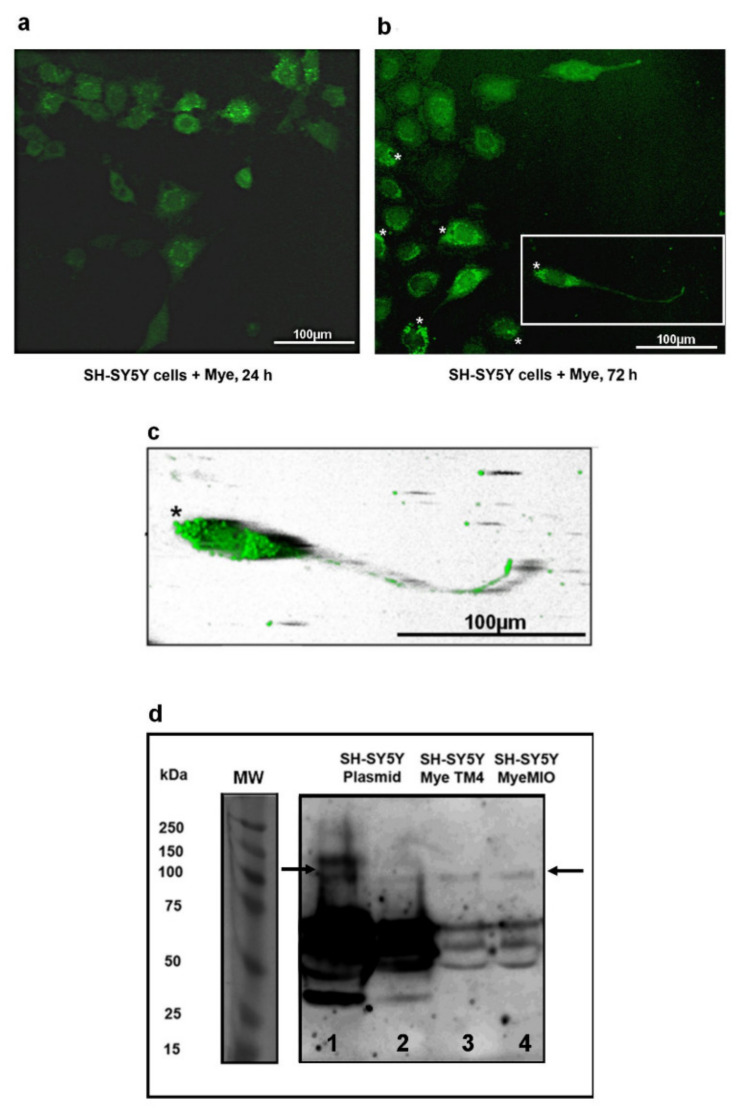
Myelinosomes from MIO-M1 cells carrying mHTT-exon1 protein are incorporated into neuronal SH-SY5Y cells. (**a**) Confocal image of live naive SH-SY5Y cells after 24 h exposure to myelinosomes carrying EGFP-mHTT-exon1. (**b**) Confocal image of live naive SH-SY5Y cells after 72 h exposure to myelinosomes carrying EGFP-mHTT-exon1. Asterisks denote the concentration of green staining. Several cells develop the axons (inset); (**c**) is a 3D reconstruction of a selection from (**b**). Asterisk denotes the concentration of green staining. (**d**) WB analysis of EGFP-mHTT-exon1 content in total cell lysates from naive SH-SY5Y cells exposed to myelinosomes emitted by TM4 (line 3) or MIO-M1 (line 4) cells using 1C2 antibody. Lines 1 and 2 show the content of the EGFP-mHTT-exon1 protein in total cell lysates of SH-SY5Y cells transfected with plasmid vector. Note the presence of SDS-soluble oligomers of the EGFP-mHTT-exon1 protein (arrows). Abbreviations: Mye MIO—myelinosomes from MIO-M1 cells; Mye TM4—myelinosomes from TM4 cells; MW—molecular weight.

We incubated myelinosomes isolated from a culture media of MIO-M1 cells expressing EGFP-mHTT-exon1 with human neuroblastoma SH-SY5Y cells. This neuronal-like cell line is commonly used in retinal research as an in vitro model for specific applications [54,55]. We monitored living SH-SY5Y cells by confocal microscopy at different time points of incubation with myelinosomes. Figure 4a shows that 24 h incubation with myelinosomes loaded with EGFP-mHTT-exon1 transformed untransfected SH-SY5Y cells into green-colored ones, suggesting the incorporation of the EGFP-mHTT-exon1 protein into naive cells. By 72 h of incubation, the green fluorescent signal became more pronounced. At this time point, bright green spots were observed in the cytoplasm (Figure 4b) and in the axons of SH-SY5Y cells (Figure 4b,c). The movement of green signal along the axons and plasma membranes of naïve SH-SY5Y cells was clearly seen by time-lapse microscopy (Supplementary Materials Movies).

To ensure the incorporation of the EGFP-mHTT-exon1 protein into naive neuronal-like SH-SY5Y cells, we analyzed cell lysates by WB [56] using 1C2 antibody against polyQ-track [57,58]. The lysates of SY-SY5Y cells transfected with plasmid vector carrying *EGFP-mHTT-exon1* were used as a control. WB analysis of control preparations (Figure 4d) demonstrated that 1C2 antibody stained 75 kDa protein bands corresponding to the EGFP-mHTT-exon1 protein (strong staining), and two bands migrating as 200 kDa proteins, which seemingly corresponded to the SDS-soluble oligomers of mHTT-exon1 [59]. Both 75 kDa and 200 kDa bands were present on the blots from the lysates of SY-SY5Y cells challenged with myelinosomes emitted by MIO-M1 cells or TM4 Sertoli cells (used for comparison) expressing EGFP-mHTT-exon1. Thus, the naive neuronal-like SH-SY5Y cells incorporate the myelinosomes carrying mutant EGFP-mHTT-exon1 protein. We then looked for the mechanism of such incorporation.

### 2.5. Macropinocytosis Does Not Support the Incorporation of Myelinosomes into Human Neuroblastoma SH-SY5Y Cells

**Figure 5:**
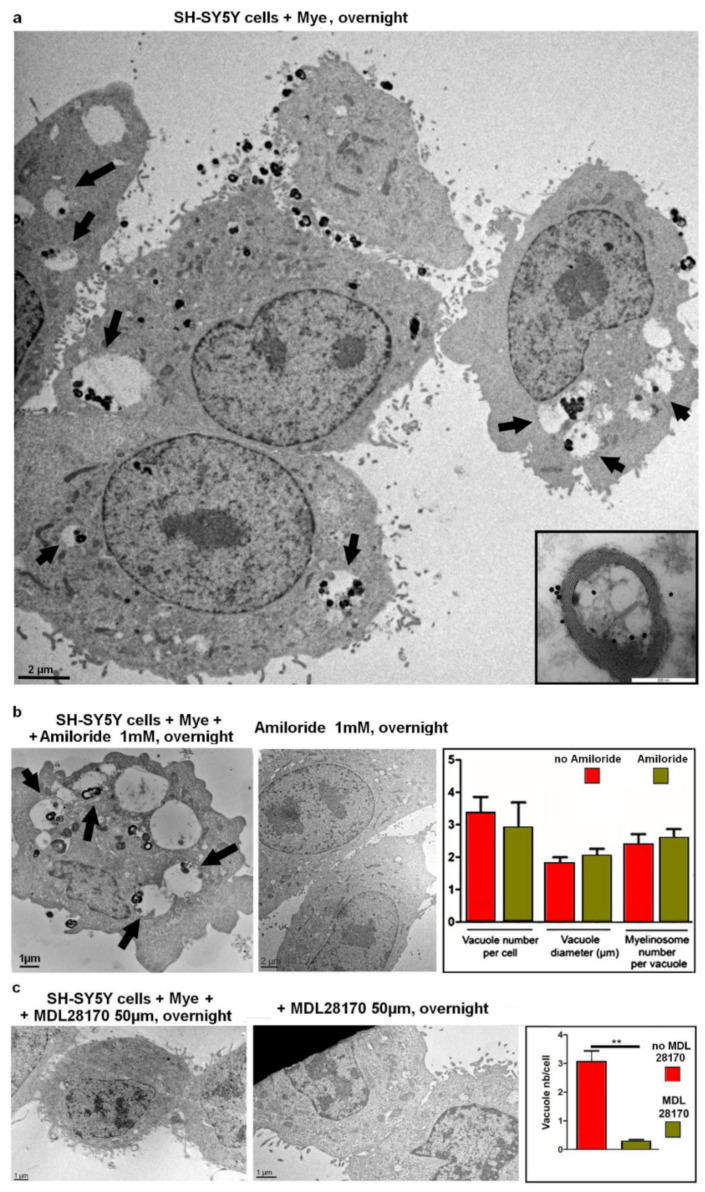
Incorporation of myelinosomes into SH-SY5Y cells is inhibited by synthetic drug MDL28170. (**a**) Electron micrograph of SH-SY5Y cells after overnight exposure to myelinosomes carrying EGFP-mHTT-exon1. Note that incorporated myelinosomes are found in cytoplasmic vacuoles (arrows). Selection in right corner is anti-EGFP immunogold labeling of the myelinosome preparation used in this experiment. (**b**) Electron micrograph showing amiloride-pretreated SH-SY5Y cells after overnight incubation with myelinosomes loaded with EGFP-mHTT-exon1 (left panel), and amiloride-pretreated SH-SY5Y cells, which were not exposed to myelinosomes (central panel). Arrows denote cytoplasmic vacuoles harboring myelinosomes. Quantification histogram (right panel) depicts the number of myelinosome-bearing vacuoles/per cell, the diameter of the vacuoles, and the number of myelinosomes per vacuole in amiloride-pretreated and untreated SH-SY5Y cells following overnight incubation with myelinosomes loaded with EGFP-mHTT-exon1. No significant variations were revealed. (**c**) Electron micrograph showing SH-SY5Y cells after overnight incubation with myelinosomes carrying EGFP-mHTT-exon1, in the presence of membrane fusion inhibitor MDL28170 (left panel) and MDL28170-pretreated SH-SY5Y cells, which were not exposed to myelinosomes (central panel). Quantification histogram (right panel) depicts the number of myelinosome-harboring vacuoles in SH-SY5Y cells in the presence or absence of membrane fusion inhibitor MDL28170. ** *p* < 0.001 by two-tailed Student’s *t* test. Abbreviations: Mye—myelinosomes.

By EM, we examined the ultrastructure of SH-SY5Y cells challenged/or not with myelinosomes carrying EGFP-mHTT-exon1. Supplementary Materials Figure S3c shows that in control preparations (no treatment with exogenous myelinosomes), no intrinsic myelinosome organelles were found in the cytoplasm of SH-SY5Y cells. On the contrary, these organelles were widely present in the cytoplasm of SH-SY5Y cells incubated with exogenous myelinosomes (Figure 5a). Remarkably, in SH-SY5Y acceptor cells, myelinosomes clustered in large vacuoles located close to the plasma membrane. The aspect and the size (ø1.8 ± 0.20 μm (*n* = 22)) of vacuoles harboring myelinosome vesicles resembled macropinosomes, which are the vacuoles formed during the macropinocytosis process [60,61]. Quantification analysis showed that each vacuole contained 2.37 ± 0.34 (*n* = 27) myelinosomes, but several vacuoles harbored up to 8 myelinosomes.

To check for the involvement of macropinocytosis in the transfer of myelinosomes into acceptor cells, we pre-incubated the naive SH-SY5Y cells with macropinocytosis inhibitor amiloride [62] and then exposed the cells to myelinosomes loaded with EGFP-mHTT-exon1. By IF examination, both amiloride-treated and untreated SH-SY5Y cells became green-colored after incubation with EGFP-mHTT-exon1-loaded myelinosomes (Supplementary Materials Figure S3d,e). EM study, followed by quantification analysis, revealed no variation between amiloride-treated and control preparations in respect to (1) relative size of vacuoles harboring myelinosomes, (2) quantity of vacuoles per cell, and (3) content of myelinosomes per vacuole (Figure 5b). Furthermore, EM examination did not reveal macropinocytosis-induced membrane riffling [63] in SH-SY5Y cells exposed to exogenous myelinosomes (Figure 5a). Thus, macropinocytosis does not support an integration of myelinosomes carrying EGFP-mHTT-exon1 in SH-SY5Y cells.

### 2.6. Incorporation of Myelinosomes into Human Neuroblastoma SH-SY5Y Cells Is Inhibited by Synthetic Drug MDL 28170

Alternatively, membrane fusion—a very fast transport process widely used in the CNS for neurotransmission—could be implicated in the incorporation of myelinosomes into acceptor cells [64,65]. In several model systems, membrane fusion is efficiently inhibited by the synthetic drug MDL28170 [66]. Therefore, we pre-incubated the naive SH-SY5Y cells with MDL28170 and then exposed them to myelinosomes loaded with EGFP-mHTT-exon1. MDL28170-treated and untreated cells were further compared using fluorescent and electron microscopy. IF microscopy showed the decrease of green signals in MDL28170-treated preparations compared to untreated ones (Supplementary Materials Figure S3f). This result was supported by EM analysis, which revealed a strong decrease of content of myelinosome-harboring vacuoles in MDL28170-treated cells: 0.28 ± 0.07 (*n* = 46) vs. 3.05 ± 0.39 (*n* = 22) in the control preparations (Figure 5c). Some rare large vacuoles observed in the cytoplasm of cells treated with MDL28170 were either free of, or contained no more than 1 myelinosome (Supplementary Materials Figure S3g).

Thus, the pre-treatment with membrane fusion inhibitor MDL28170 prevented incorporation of EGFP-mHTT-exon1-loaded myelinosomes in the cytoplasm of acceptor SH-SY5Y cells. This indicates that a membrane fusion mechanism likely contributes to the transfer of myelinosome-bound EGFP-mHTT-exon1 into naïve neuronal-like cells.

## 3. Discussion

As a part of the CNS, the retina is termed a “window” to the brain because it exhibits similar principles of cellular organization and metabolism [1]. Therefore, the retina may provide a useful model to characterize the mechanisms leading to neuronal pathology in HD. In this work, we undertook an EM examination of the retina of transgenic HD R6/1 mice. We show for the first time that the pathological retina of HD mice is enriched with unusual organelles myelinosomes. Being loaded with mutant mHTT-exon 1, myelinosomes can be secreted from glial Müller cells and further incorporated into neuronal cells through a membrane fusion mechanism, thereby contributing to HD spreading.

In most retinal degenerations triggered by gene mutations or environmental factors, photoreceptors are the primary target [67]. The destruction of the outer segment is typically followed by photoreceptor cell death and then by a profound remodeling of neural retina remnants [68]. Compared to other types of retinal pathologies, R6/1 mice retina shows an atypical pattern of degeneration, in which the destruction of photoreceptor OS does not lead to massive death in the ONL or to significant morphological abnormalities in the inner retinal neurons. In contrast to retinal phenotype in R6/2 mice, wherein apoptotic cell loss was prominent in the outer retina [23], the apoptotic photoreceptor nuclei were extremely rare in R6/1 ONL, and no shrinking of rod synaptic contacts with retinal interneurons (rod spherules) was observed in the OPL. In line with this, the pattern of retinal degeneration in HD R6/1 mice rather resembled those previously described for the retina of spinocereballar ataxia (SCA7) mice [69]. In SCA7 mice, the CAG expansion affects the gene of *ataxin-7*, coding for the ataxin 7 protein, a component of the STAGA transcription coactivator complex [70]. The presence of ectopic synapses in the ONL due to infiltration of the neurites from retinal interneurons, and a decrease of ERG amplitude, were characteristics of both R6/1 and R6/2 retinas as well as other retinal pathologies [69,71,72,73,74]. As in R6/2 mice, the inner retina of R6/1 mice, including the nuclei of interneurons, IPL and ILM looked preserved and did not manifest ultrastructural abnormalities [23]. While upregulation of GFAP in R6/1 Müller cells was reported previously [25], we revealed no morphological signs of Müller cell stress by EM analysis.

A striking peculiarity of R6/1 retina was the ubiquitous presence of unusual organelles myelinosomes, which were detected in all nuclear and plexiform layers, as well as in the subretinal space. Formed of stacked electron-dense osmiophile membranes enwrapping the cavity filled with an electron lucid matrix, in ultra-thin cross-sections myelinosome organelles have a myelin sheath structure insulating nerve cell axons. This complicates the detection of myelinosomes in micrographs from brain sections, but not from the retinal ones, because the axons of retinal neurons are not insulated with myelin sheaths [75]. Various extra-CNS tissues devoid of myelin as testis seminiferous tubules, trachea and others, or myelin-free retinas from different pathological and non-pathological species have been reported to produce myelinosomes [43,76,77,78,79,80,81,82,83,84]. Various names have been given to these organelles, which are also known as multimembranous bodies, multilamellar bodies, myelin bodies, myelin figures, myelin-like organelles, membranous bodies, vesiculated membranes, or zebra bodies, thereby confusing the identification of myelinosomes in different normal and pathological tissues [45]. Interestingly, myelinosomes were also detected in the retina of SCA7 mice, though they were termed as vesiculated membranes [85].

EM analysis of HD R6/1 retina showed that the retinal pool of myelinosomes was formed by both neurons and glia. Noteworthy is the fact that myelinosome-containing cells did not exhibit any morphological signs of necrotic or apoptotic transformations. In photoreceptor and retinal interneurons, myelinosomes displayed both intracellular and extracellular location, being encountered in perinuclear cytoplasm, sometimes in close proximity/association with ER. Myelinosomes were also present in the synaptic terminals of photoreceptors as well as in the neuronal processes, indicating the translocation of myelinosomes across the cytoplasm of retinal neurons. Moreover, EM analysis revealed that the glial Müller cells produced and secreted myelinosome organelles, which were further found in the subretinal spaces. Collectively, this suggests the ability of myelinosomes to move across the R6/1 retina, and probably to exchange between different cell populations.

The origin and function of myelinosomes in different tissues remain unclear. The possible derivation of myelinosomes from nuclear membrane or from the membranes of ER is still debated [44,86]. As recently suggested, in the CNS, myelinosomes are the result of myelin breakdown [46]. This is not the case in the retina, because retinal neurons are not myelinated, and Müller glia does not produce myelin complex [75]. We previously showed that myelinosomes secreted by testis Sertoli cells were free of main extracellular vesicle marker tetraspanin CD63 protein [43,87]. In R6/1 mice, testis and in Sertoli cells transfected with *EGFP-mHTT-exon1* vector myelinosomes were loaded with mHTT-exon1 [43]. In this work, we showed that myelinosomes emitted from the glial human MIO-M1 Müller cells, transfected with *EGFP-mHTT-exon1* vector, also carry the EGFP-mHTT-exon1 protein. It should be noted that myelinosomes from control preparations were free of normal EGFP-HTT-exon1. The role of these “empty/unloaded” myelinosomes remains unclear. Therefore, it seems likely that, in a pathological situation such as HD, myelinosome organelles are involved in the evacuation of metabolic “waste” (mutant protein), thereby contributing to proteostasis maintenance in several tissues, at least in the retina and in the testis [43,44]. Noteworthy is that we previously showed that myelinosome organelles also evacuated another mutant protein responsible for cystic fibrosis (CF), F508delCFTR [43]. In relation to this, the potential impact of ”wasting” myelinosomes on neighboring cells is of particular interest in pathological situations when normal evacuation routes could be compromised due to pathological processes.

The presence of EGFP tag gave us an opportunity to follow the fate of green-colored myelinosomes, carrying mHTT-exon1 after their incubation with the naive neuronal-like SH-SY5Y cells. Time-lapse recording showed that living neuronal cells incorporated extrinsic myelinosomes, which moved in perinuclear cytoplasm and then translocated toward the growing axon. Of note is that the transneuronal propagation of mHTT via synaptic contacts was also evidenced in the HD CNS. This occurs in an anterograde direction and inversely correlates with synaptic plasticity [88,89]. In relation to this, time-lapse recording supported our in situ observations, which revealed myelinosomes in different parts of retinal neurons, including synaptic terminals. The presence of myelinosomes loaded with mHTT in the synaptic terminals of retinal neurons might be one of the factors contributing to the disruption of synaptic function in R6/1 HD retina [22,23,24,25,26,88].

In our in vitro model, the incorporation of myelinosomes carrying mHTT-exon1 by neuronal-like cells was blocked after pharmacological inhibition of membrane fusion by means of the synthetic drug MDL28170. Membrane fusion is a mechanism widely used in the CNS for neurotransmission [64,65,89]. Interestingly, inhibition of membrane fusion by botulinum toxin was shown to block transneuronal transfer of mHTT via synaptic contacts [16]. We demonstrate here that SH-SY5Y cells uses membrane fusion to incorporate the extrinsic myelinosomes, so that the mHTT-exon1 protein was clearly detected in cell lysates by WB. Moreover, SDS-soluble oligomers of mHTT-exon1, which are believed to confer synaptic dysfunction [59,90], were also present in recipient cells, suggesting an intracellular processing of myelinosomes and/or their cargoes. Remarkably, whatever the origin of myelinosomes carrying mHTT-exon1 (Müller or Sertoli cell-derived), the mutant protein was delivered in recipient cells. Therefore, the inter-cellular exchange of myelinosomes derived from different cells/sources inside the tissue seems to be plausible. This corroborates the current view on different ways of mHTT propagation, including those via the bloodstream. The later was recently reported to disseminate the disease in parabiosis experiments [91].

The abundance and ubiquitous presence of myelinosomes in HD retina argued for their significance for this pathology. Interestingly, myelinosomes were not revealed in R6/2 retinas, while some “curled structures” were noticed in R6/2 subretinal space [23]. Nevertheless, myelinosome-like osmiophile-stacked structures were detected in situ in brain striatal neurons from R6/2 HD model mice, being termed as lamellar lysosomes [92]. We previously demonstrated that myelinosomes, loaded with the mHTT-exon1 protein were abundant in pathological R6/1 HD testis [43]. Of note is that myelinosome-like organelles were recently found in human seminal plasma, suggesting that myelinosomes and/or their cargoes can be evacuated through biological fluids [93]. Much like somatic Sertoli cells from the testis, retinal Müller glia exhibits high secretory activity and plays a critical role in the removal of metabolic wastes [38,94]. Therefore, one can speculate that evacuation of retinal myelinosomes, loaded with mHTT-exon1, would proceed through vitreous body and probably through other ocular fluids. This seems plausible because mHTT was found in the cell-free preparations of biological fluids such as blood and CSF [91,95]. In relation to this, the analysis of ocular fluids for the presence of myelinosomes could be suggested as an additional HD diagnostic test. Current eye dissection protocol for EM examination of the retina requires complete removal of the vitreous body, as well as of the aqueous humor. Thus, other protocols are needed for further analysis of ocular fluids to detect myelinosome organelles as possible HD biomarkers [96].

Müller cells are the only macroglial cells of the retina, and they insure many of the functions carried out by brain oligodendrocytes and astrocytes [97]. Brain glia is known to influence HD phenotype, exerting either deleterious or beneficial effect. Recent study on the HD *Drosophila* model showed that glia acted as an obligatory intermediate in mHTT spreading between synaptically-connected neurons [98,99]. Study from a rodent model demonstrated that the neonatal chimerization with normal glia delayed disease progression in R6/2 transgenic HD mice. Therefore, glial cell replacement was proposed for HD treatment [100]. Conversely, engrafting with glia expressing polyQ-expanded mHTT induced neurophysiological abnormalities [100]. The mechanism of glial-derived effects remains obscure. In *Drosophila* models, the phagocytic properties of glia are discussed [97,98]. In mammalian models, the view on exosome-mediated transfer of mHTT predominates, because mHTT was found in the exosome fraction from patient fibroblasts, from induced pluripotent stem cells and from human neuroblastoma cell line SH-SY5Y transfected with GFP-mHTT-exon1 [101]. Injection of this fraction in recipient animals or in recipient cells induced HD spreading [101]. Nevertheless, exosome-mediated transfer does not explain the phenomenon of HD glial toxicity because no mHTT protein was found in the isolated exosome fraction from HD glial cells [102]. In relation to this, one of the possible explanations of the deleterious effect of HD glia could be related to myelinosome organelles loaded with the toxic mHTT protein and secreted outwards.

Summing up, here we demonstrate the presence and possible role of myelinosome organelles in HD retina at the middle symptomatic stage of the pathological process. A widespread distribution of myelinosomes suggests their importance for HD retina at this stage of disease development. To date, we do not know how early myelinosomes arise in HD retina. Another issue to address is whether myelinosome emergence correlates with the severity of the pathological process. If so, exploring the ocular fluids for the presence of myelinosomes could reveal a reliable biomarker to assess disease progression in HD individuals. Taking into consideration a systemic pattern of HD, which affects, among other things, the reproduction axis, exploring the seminal fluid for myelinosome presence seems to be a promising tool for HD diagnostic in pre-manifesting individuals. To date, electron microscopy/cryo-electron microscopy [93] techniques are only available to detect myelinosomes in biological fluids. In relation to this, one of our current challenges is to develop routine methods for myelinosome detection using nanotechnology approaches such as the coupling of myelinosomes with gold nanoclusters, and others. Exploring the fundamental aspects of myelinosome biogenesis, characterization of the molecular fingerprints of these organelles and the molecular mechanism of myelinosome-mediated sorting and transfer of mHTT-exon1 are among further research directions aimed at developing novel therapeutic strategies for HD treatment.

## 4. Materials and Methods

### 4.1. Animals and Cells

Heterozygous R6/1 mice were maintained at LNCA (Strasbourg, France) on C57BL/6J genetic background. Animal studies were conducted in accordance with French regulations (EU Directive 2010/63/UE–French Act Rural Code R 214-87 to 126). The animal facility was approved by veterinary inspectors (authorization no. E6748213) and complies with the Standards for Human Care and Use of Laboratory Animals of the Office of Laboratory Animal Welfare. The procedures were approved by a local ethics committee (CREMEAS) and the French Research Ministry (no. APAFIS#504-2015042011568820_v3). Mice were housed in a controlled-temperature room maintained on a 12h light/dark cycle. Food and water were available ad libitum. Mice were genotyped as described in [27]. Wild type littermate mice came from the same breeding colony. Mice were killed by cervical dislocation, and their eyes were rapidly enucleated for TEM analysis (see below).

Human MIO-M1 cells were kindly provided by Professor Astrid Limb (University College London, UK); human neuroblastoma SH-SY5Y cells were from the ATCC Cell Biology Collection (ATCC Molsheim, France), murine TM4 Sertoli cells were from the IGBMC (Illkirch, France) cell culture service collection. MIO-M1 and SH-SY5Y cells were cultured in DMEM (GIBCO, 31966,Thremo Fisher, Waltham, MA, USA) medium supplemented with 10% FCS and 50 μg/mL gentamycin (P06-03025P, PAN Biotech, Wimborne, UK) and 2 μg/mL fungizone (A2411, Sigma Aldrich, St. Louis, MO, USA). TM4 Sertoli cells were cultured in DMEM/F12 medium (Gibco, number 31330), containing 2.5% FBS and 5% HS and were supplemented with 1% antibiotic mix (10,000 U/mL penicillin and 50 mg/mL streptomycin, GIBCO 15140). In all cases, 0.5 × 10^6^ cells were plated into 6-well dishes or in 3.5 cm^2^ petri dishes and cultured at 37 °C with 5% CO_2_. All cell lines were tested for mycoplasma contamination using a mycoplasma detection kit (LT07-418, LONZA, Basel, Switzerland).

### 4.2. Chemicals and Plasmids

Dulbecco’s phosphate buffered saline (PBS) (number D5652), bovine serum albumin (BSA) (number A9647), amiloride (number A-7410), and MDL28170 (number M6690) were provided by Sigma-Aldrich, St. Louis, MO, USA. The polyclonal rabbit primary antibodies were anti-EGFP (Molecular Probes, A6455, Thermo Fisher, Waltham, MA, USA). The monoclonal mouse antibodies used were anti-GFP (B-2) (sc-9996, Santa-Cruz Biotechnology, Dallas, TX, USA), 2B4, and 1C2 (IGBMC antibody platform, Illkirch, France). The other antibodies were secondary anti-rabbit (sc-2004, Santa-Cruz Biotechnology, Dallas, TX, USA) and anti-mouse (NA931, GE Healthcare, Bath, UK) antibodies conjugated to horseradish peroxidase; for immunostaining: anti-rabbit and anti-mouse antibodies conjugated to Alexa-488 (CAR-A21441), to Alexa-594 (GAM- A21422, DAM-A31570) from Molecular Probes (Thermo Fisher, Waltham, MA, USA). For immunoelectron microscopy, anti-rabbit antibodies conjugated with 15 nm gold beads (Auroprobe TM/EM, LRPN422V/AA/G15, Auroprobe, New Delhi, India), and anti-mouse antibodies conjugated with 10 nm gold beads (G7777, Sigma-Aldrich, St. Louis, MO, USA) were used. The vectors used for HTT and mHTT expression were pEGFP-C2-tr/HTT-17Q and pEGFP-C2-tr/HTT-142Q, generated at IGBMC (Strasbourg, France).

### 4.3. Transient Transfection

Transfection of MIO-M1, SH-SY5Y and TM4 cells with expression vectors were performed using Lipofectamine 2000 (Invitrogen, Thermo Fisher, Waltham, MA, USA) reagent in 6-well plates or in 3.5 cm^2^ petri dishes, containing 0.5 × 10^6^ cells following the manufacturer’s instructions. The ratio of plasmid/Lipofectamine in 2.0 mL of serum-free incubation mix was 4.0 μg/10 μL for MIO-M1 and SH-SY5Y cells and 4.4 μg/16 μL for TM4 cells transfected with vectors coding for EGFP-HTT-exon1 and EGFP-mHTT-exon1. Unless otherwise noted, cells were used for further manipulations 48 h post transfection.

### 4.4. Differential Centrifugation of Culture Media

Culture media was collected from 48 h post-transfection MIO-M1 and/or TM4 cells expressing EGFP-HTT-exon1/EGFP-mHTT-exon1 and then centrifuged at 600× *g* for 10 min. The pellet was collected; the supernatant was centrifuged again at 20,000× *g* for 90 min. The 20,000× *g* pellets were further used for EM, immuno-EM, and for naive cell treatment.

### 4.5. Treatment of Cells with Myelinosomes

Myelinosomes were collected from 48 h post-transfection culture media of MIO-M1 and/or TM4 cells expressing EGFP-mHTT-exon1 and added to non-transfected SH-SY5Y cells. Routinely, myelinosomes were collected from 2 wells of transfected cells, and the purified myelinosomes were added into 1 well of naive cells. Naive cells were incubated with myelinosomes at 37 °C with 5% CO_2_. In EM experiments, the presence of EGFP-mHTT-exon1 in myelinosome preparations was checked by immuno-EM.

### 4.6. Treatment of Cells with Inhibitors of Macropinocytosis and Membrane Fusion

SH-SY5Y cells were pre-incubated at 37 °C for 1 h with amiloride (1 mM) or MDL28170 (50 µM or 100 µM), then loaded with myelinosomes carrying EGFP-mHTT-exon1. Live cells were imaged using fluorescent microscope (see below) at different time points of incubation with myelinosomes. The next day, the culture media was removed and the cells were washed with PBS and then processed for EM.

### 4.7. Western Blotting

Proteins were revealed in cell extracts obtained after lysis of 10^7^ MIO-M1, SH-SY5Y, or TM4 cells growing in 6-well dishes. Cells were washed with cold PBS and then collected by scraping on ice in a lysis buffer (50 µL/per well) containing 50 mM Tris-HCl, 150 mM NaCl, 1 mM EDTA, 1% Triton X-100, phosphatase inhibitor cocktail (04906845001, Roche Applied Science, Penzberg, Germany), and protease inhibitor cocktail (11697498001, Roche Applied Science, Penzberg, Germany). Then, 17 µL 4X Laemmli sample buffer was added, and the samples were incubated at 99 °C for 10 min. Proteins were separated onto 8% SDS-polyacrylamide gels, then transferred (2 h, 150 V) to nitrocellulose membranes (0.20 mm pore). Blots were blocked for 2 h in TBS containing 5% (*w*/*v*) non-fat dry milk. Blots were incubated with 1C2 (1/1000) antibodies in TBS containing 0.5% (*w*/*v*) non-fat dry milk at 4 °C overnight. Membranes were then incubated with peroxidase-conjugated anti-rabbit (1/5000) or anti-mouse (1/5000) antibody in blocking buffer for 1 h at room temperature. Immunoblots were developed with chemiluminescence system Luminata Forte Western (WBLUFO100, Millipore, Molsheim, France). In some cases, the blots were stripped in 25 mM Glycine-HCl buffer containing 1% *w*/*v* SDS, pH 2 for 30 min; membranes were then washed with TBS, blocked, and re-probed as above.

### 4.8. Immunocytochemistry

Indirect immunofluorescence was performed following conventional procedures. Briefly, cells growing on 3.5 cm^2^ petri dishes were fixed in paraformaldehyde/PBS (pH 7.4), permeabilized with 0.5% Triton X-100/PBS, then incubated overnight at 4 °C with primary antibodies in 1% BSA/PBS; the dilution of antibodies was as follows: 1:500 for anti-EGFP, and 1:250 for 2B4 and anti-1C2 antibodies. The dilution of the secondary antibodies was 1:500 in 1% BSA in PBS. Cell nuclei were stained with DAPI (4′,6 diamidino–2-phenylindole; 0.5 μg/mL). Slides were observed under an inverted microscope DMT 6000 Leica (Leica Microsystems, Wetzlar, Germany). The pictures were taken from a DFC350FX camera (Leica Microsystems).

### 4.9. Confocal Microscopy, Videomicroscopy, and Image Analysis

Samples were examined by confocal laser scanning microscopy using a confocal FV-1000 station installed on an inverted microscope IX-81 (Olympus, Rungis, France). Multiple fluorescence signals were acquired sequentially to avoid crosstalk between image channels. Fluorophores were excited with a 405 nm diode (for DAPI), the 488 nm line of an argon laser (for AF488), and the 543 nm line of a HeNe laser (for AF594. The emitted fluorescence was detected through spectral detection channels between 425 to 475 nm, 500 to 530 nm, and 555 to 655 nm for blue, green, and red fluorescence, respectively. Objective lens ×10, ×20 (Olympus) or Olympus UplanSapo ×60 oil, 1.4 NA were used to obtain maximal resolution. When necessary, optical sectioning of the specimen (Z series) was driven by a *Z*-axis stepping motor through the entire thickness of the cell layer and analyzed with Imaris 3D software (Bitplane, Oxford instruments, Oxfordshire, UK).

Videomicroscopy was carried out with the same confocal laser microscope using ×40 oil UAPO NA 1.30 with 488 nm laser illumination for green fluorescence detection (500-600 nm). Green fluorescent images were acquired at the rate of 1 image per second 85 times, at 5 frames per second corresponding to a 17 s movie.

### 4.10. Electron Microscopy Studies

Whole eyes from 23-week-old R6/1 and control CBAxC57BL/6 mice (10 eyes for each preparation) were enucleated and then pierced with a thin needle in the cornea and fixed by immersion in 2.5% glutaraldehyde (Sigma-Aldrich, G5882) and 2.5% formaldehyde in cacodylate buffer (0.1 M, pH 7.4), as in [68]. Ten minures after, the lens and the cornea were removed and the eyecups were fixed overnight in the same fixative, then washed in cacodylate buffer for 30 min. Eyes were then post-fixed in 1% osmium tetroxide (201030, Sigma-Aldrich, St. Louis, MO, USA) in 0.1 M cacodylate buffer for 1 h at 4 °C, dehydrated through graded ethanol (50%, 70%, 90%, and 100%), and embedded in Epon 812. Cells and/or 20,000 g culture media pellets from MIO-M1 and/or TM4 cells expressing EGFP-mHTT-exon1 or EGFP-HTT-exon1 were fixed and dehydrated through the same protocol. Ultrathin sections of 70 nm were cut and contrasted with uranyl acetate and lead citrate and examined at 80 kV with a JEM1010 electron microscope (Jeol, Tokyo, Japan) or at 120 kV with a JEOL 1400 electron microscope (Jeol, Tokyo, Japan). Images were captured digitally by a Quemesa, Olympus Systems Imaging Solution Camera (Olympus, Rungis, France) or by an Orius 1000 Gatan camera (Gatan, Warrendale, PA, USA).

For immunolabeling, 100 nm sections adhered onto nickel grids were used. Sections were oxydated for 30 min in 5% sodium metaperiodate/bidistilled H_2_O, then washed for 3 × 5 min in bidistilled H_2_O and blocked in 3% BSA/PBS for 30 min. The incubation with primary anti-GFP or 2B4 antibodies (dilution 1/200 in PBS) was performed overnight at 4 °C. The sections were then washed for 3 × 5 min in 0.05% Tween/PBS and incubated with secondary anti-rabbit antibodies (dilution 1/100) conjugated to 15 nm colloidal gold for 1 h at room temperature. After washing, the sections were contrasted with uranyl acetate and lead citrate and examined at 80 kV with a JEM1010, Jeol Electron Microscope (Jeol, Tokyo, Japan) equipped with a Quemesa, Olympus Systems Imaging Solution Camera (Olympus, Rungis, France).

### 4.11. Statistical Analysis

The count of myelinosomes were performed on 300 µm^2^ randomly chosen areas in EM grids containing the preparations of 20,000 g culture media pellet fractions from MIO-M1 cells expressing EGFP-HTT-exon1 and EGFP-mHTT-exon1. Two-tailed Student’s *t* test for unpaired data was used for single comparisons between different experimental groups. Differences were considered statistically significant for a value of *p* < 0.05 and *p* < 0.001.

## 5. Conclusions

The retina is increasingly recognized as a useful model to characterize the mechanisms leading to neuronal pathologies [1]. The widespread presence of unusual organelles myelinosomes in all neuronal and glial cell populations, as well as in extracellular spaces, suggests the importance of myelinosomes for pathological HD retina. Exploring the mechanism of myelinosome-mediated transfer of mHTT-exon1 can be instrumental in developing novel therapeutic strategies to slow mHTT spreading over the CNS, while analysis of ocular fluids for myelinosome presence could reveal an additional biomarker for HD diagnostics.

## Supplementary Materials

The following are available online at https://www.mdpi.com/article/10.3390/ijms222312771/s1
