# Supplementary material for: Myelinosome Organelles in the Retina of R6/1 Huntington Disease (HD) Mice: Ubiquitous Distribution and Possible Role in Disease Spreading
Source: Int J Mol Sci. 2021 Nov 25;22(23):12771. doi: 10.3390/ijms222312771 (PMC8657466; doi:10.3390/ijms222312771)
Supplement: Supplementary file 1 [file ijms-22-12771-s001.zip › ijms-1440919 supp.pdf]

Legends for Supplementary Figures and Movies.

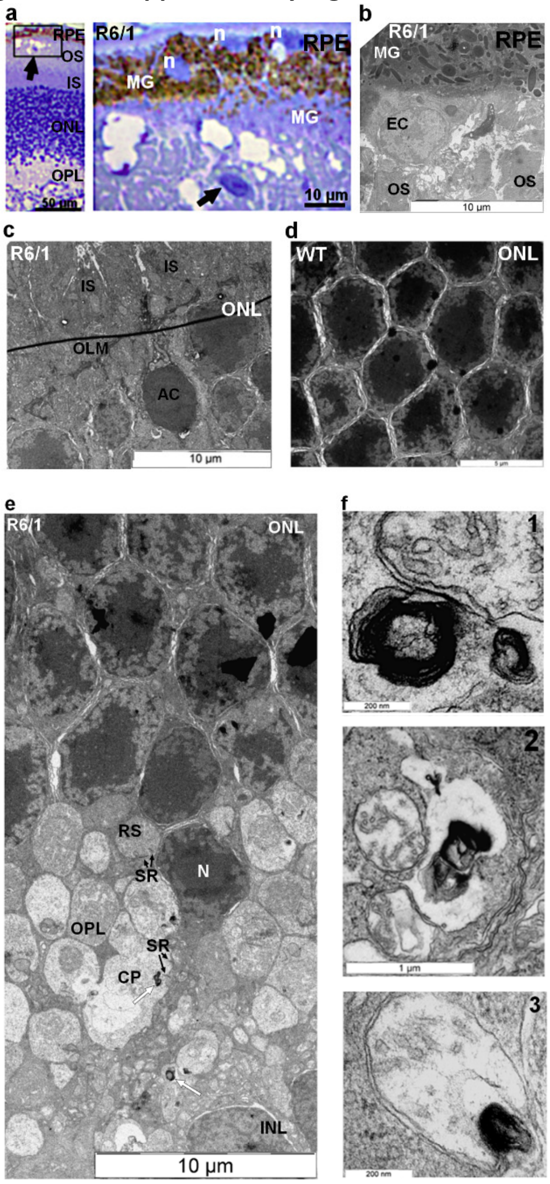

Suppl.1

Supplementary Figure S1. Histological and electron microscopy examination of 23-week-old R6/1 retina. (**a**) Histological section of 23 week-old outer retina from R6/1 mouse, showing an ectopic cell (arrow) and melanin granules in the subretinal space. Note the irregular pattern of nuclear packaging

in RPE layer. Right panel is a selection from left panel. **(b)** Electron micrograph of the outermost part of R6/1 retina, containing RPE cells and the fragments of degenerative OS. Note that the ectopic cell (EC) from subretinal space is not engulfed by RPE. **(c, d)** Electron micrograph of the fragment of the outer retina of R6/1 **(c)** and control **(d)** mice. **(c)** shows photoreceptor IS, which are not adjacent to each other, and an apoptotic photoreceptor nucleus located below the intact OLM. Note the absence of myelinosomes in the vicinity of the apoptotic nucleus. **(e)** Electron micrograph showing a displaced photoreceptor cell in OPL of R6/1 retina, containing the synaptic terminals of rod and cone photoreceptors. Note an electron-dense myelinosome (white arrow) located in cone pedicle (CP) next to synaptic ribbons (black arrows). Another myelinosome is located close to INL. **(f)** Magnifications of myelinosomes (1, 2, 3) from Fig. 2 a. Note the osmiophile membranes, enwrapping an electron-lucid matrix.

Abbreviations: CP - cone pedicle; INL-inner nuclear layer; IS – photoreceptor inner segment; MG-melanin granules; n-nucleus; OLM-outer limiting membrane; ONL – outer nuclear layer; OPL- outer plexiform layer; RPE – retinal pigmented epithelium; SR-synaptic ribbon.

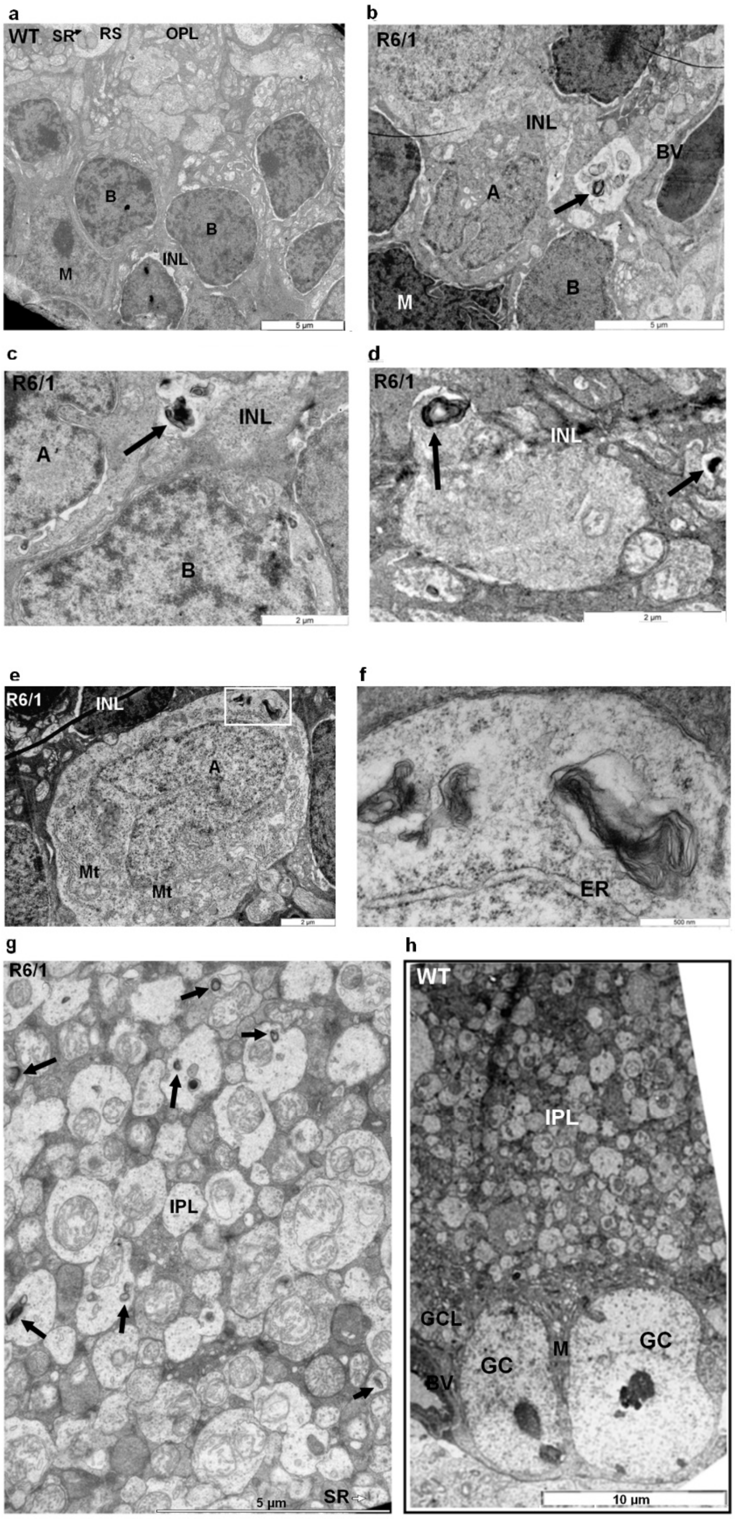

Suppl.2

Supplementary Figure S2. Electron microscopy examination of the inner retinas of 23-week-old R6/1 mice. (**a**,**b**–**f**) Electron micrograph of the INL of control **(a)** and R6/1 **(b-f)** retinas. Cell nuclei in INL are identified according to [93] **(b,c)** depict myelinosomes (black arrows) close to amacrine cell nuclei. **(d)** shows an intracellular myelinosome (black arrow) in electron-lucid cytoplasm of horizontal cell. **(e, f)**

intracellular myelinosome in the cytoplasm of amacrine cell is in close association with the membranes of ER, alike we previously reported for testis Sertoli cells [34] **(f)** is a selection from **(e).** **(g)** IPL of R6/1 retina containing a synaptic ribbon (SR). Electron-dense myelinosomes (black arrows) localize in neuronal processes formed by the dendrites of ganglion cells and by the axons of retinal interneurons. **(h)** IPL and ganglion cell layer (GCL) of control retina. Ganglion cells (GC) are separated by electron-dense processes of Müller cells (M).

Abbreviations: A - amacrine cell; B - bipolar cell; BV - blood vessel; ER- endoplasmic reticulum; H-horizontal cells; GC – ganglion cell; GCL – ganglion cell layer; INL – inner nuclear layer; M - Müller cell; SR – synaptic ribbon.

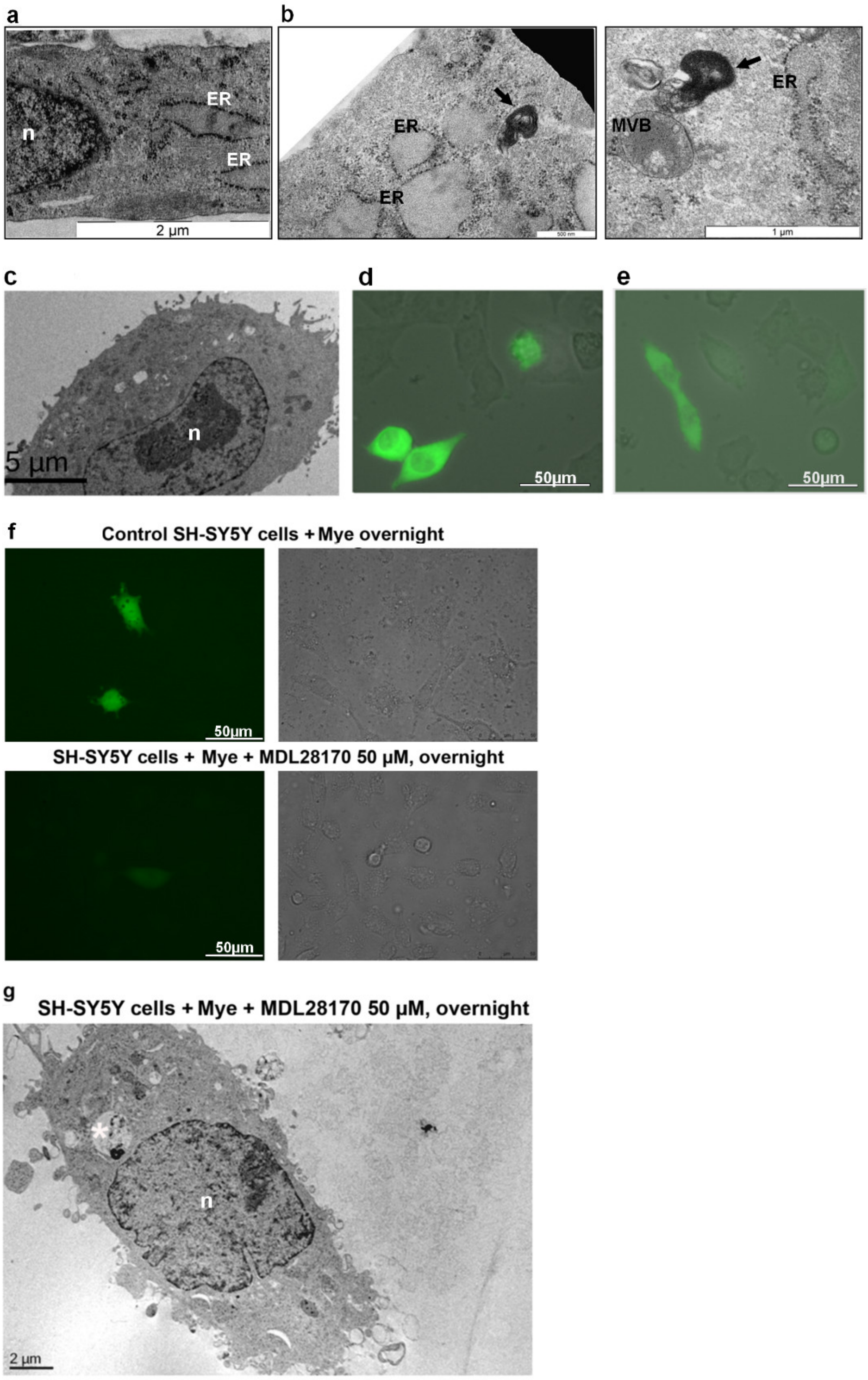

Suppl.3

Supplementary Figure S3. Electron microscopy and immunofluorescence analysis of glial Müller MIO-M1 cells expressing EGFP-mHTT-exon1 and neuroblastoma SH-SY5Y cells challenged with myelinosomes under different treatment conditions. **(a, b)** Electron micrograph of MIO-M1 Müller cells transfected **(b, right and left panels)** or not **(a)** with EGFP-mHTT-exon1. Electron-dense myelinosomes

(arrows) are next to ER membranes in **(b). (c)** Electron micrograph of control SH-SY5Y cell with no intrinsic myelinosomes. **(d, e)** IF pictures of amiloride treated **(d)** or untreated **(e)** SH-SY5Y cells after overnight incubation with myelinosomes loaded with EGFP-mHTT-exon1. Note that in both cases the cells became green-coloured. **(f)** Control (upper panel) or MDL 28170-treated (bottom panel) SH-SY5Y cells exposed overnight to myelinosomes loaded with EGFP-mHTT-exon1. Right panels are the DIC projections of left panels for better visualization of cell preparations. **(g)** Electron micrograph of MDL 28170-treated SH-SY5Y cells exposed overnight to myelinosomes loaded with EGFP-mHTT-exon1. Note the presence of single vacuole harboring a unique myelinosome (asterisk).
Abbreviations: ER-endoplasmic reticulum; n- nucleus; Mye - myelinosomes; MVB – multivesicular body.

Supplementary Movie. Migration of green staining in SH-SY5Y cells exposed to myelinosomes carrying EGFP-mHTT-exon1. Videomicroscopy recording of SH-SY5Y cells exposed to myelinosomes carrying EGFP-mHTT-exon1 observed with confocal laser microscope using x40 oil UAPO NA 1.30 with 488 nm laser illumination for green fluorescence detection (500-600 nm). Green fluorescent images were acquired at the rate of 1 image per second 85 times, at 5 frames per second corresponding to a 17 second movie.
